# Day-time variation of serum periostin in asthmatic adults treated with ICS/LABA and adults without asthma

**DOI:** 10.1186/s13223-017-0182-0

**Published:** 2017-02-08

**Authors:** Rachel Caswell-Smith, Terrianne Cripps, Thom Charles, Alexander Hosking, Meghana Handigol, Cecile Holweg, John Matthews, Mark Holliday, Corentin Maillot, James Fingleton, Mark Weatherall, Richard Beasley, Irene Braithwaite, On behalf of the Periostin Study Team

**Affiliations:** 1 Medical Research Institute of New Zealand, Private Bag 7902, Wellington, 6242 New Zealand; 2 Victoria University of Wellington, Wellington, New Zealand; 3 University of Auckland, Auckland, New Zealand; 4 Genentech Inc, San Francisco, CA USA; 5 University of Otago, Wellington, New Zealand; 6 Capital & Coast District Health Board, Wellington, New Zealand

**Keywords:** Asthma, Biomarker, Daytime variation, Periostin

## Abstract

**Background:**

We aimed to determine the effect of sampling time during the day on serum periostin levels in adult participants with and without asthma.

**Methods:**

Serum periostin was measured at 2-h intervals from 0800 to 1800 h in 16 adult participants with stable asthma prescribed inhaled corticosteroid and long-acting beta-agonist therapy, and in 16 otherwise healthy participants without asthma. Mixed linear models were used to compare time zero (08:00 h) with subsequent measurement time for serum periostin for both groups.

**Results:**

In both asthma and non-asthma, the mean (SD) serum periostin levels continuously reduced during the day from 53.5 (13.6) ng/mL at 0800 h to 50.9 (13.4) ng/mL at 1800 h (difference log periostin −0.05, P ≤ 0.001) and 50.5 (13.0) ng/mL at 0800 h to 46.2 (11.5) ng/mL at 1800 h (difference log periostin −0.08, P ≤ 0.001) respectively.

**Conclusions:**

Periostin values are higher in the morning compared with the afternoon in asthmatic and non-asthmatic adults. The small magnitude of the variation in serum periostin levels suggests that the time of day in which the serum periostin measurements are made is unlikely to influence treatment decisions if a specific serum periostin level is used to predict treatment responsiveness.

*Trial registration* Australia New Zealand Trials Registry (ACTRN12614000072617)

**Electronic supplementary material:**

The online version of this article (doi:10.1186/s13223-017-0182-0) contains supplementary material, which is available to authorized users.

## Background

Asthma is a disease with an increasing global burden [1, 2]. Appropriate biomarkers may assist with determining clinical phentoypes, predicting treatment responsiveness, and directing personalized therapy [3, 4]. Currently recognized biomarkers include fraction of expired nitric oxide (FeNO), induced sputum and blood eosinophil levels, and total and specific IgE [3, 4]. Recently serum periostin, a matricellular protein generated by airway epithelial cells and partly regulated by IL-13, has been proposed as a biomarker with a potential clinical role in severe asthma [5–8]. Serum periostin is a marker of type 2 inflammation, and has a stronger association with airways eosinophilia in severe asthma than blood eosinophil levels and FeNO [6]. High serum periostin levels may also predict responsiveness to monoclonal antibody therapy directed against IL-13 [9] IgE [10] and IL-4Ra [11]. This has led to the consideration of periostin as a predictive biomarker to identify patients most likely to respond to such monoclonal antibody therapies.

Recently we determined the reference ranges for serum periostin in two populations, an adult population without asthma or COPD [12], and an adult population with symptoms of airflow obstruction, predominantly adults with diagnosed asthma [13]. Clinical interpretation would be enhanced further by determining any time-related variation in periostin measurements, particularly within the typical timeframe covered by clinic-based assessments in primary or specialist health practice. Time-related variations are reported for other type-2-related biomarkers in asthma such as sputum and blood eosinophils [14–18], and FeNO [19–21]. Measurements of these biomarkers are generally higher overnight and in the morning than in the afternoon.

We estimated the time-related variation in serum periostin levels in adults with asthma receiving maintenance ICS and long-acting beta agonist (LABA) therapy; the clinical population in which serum periostin levels are a predictor of responsiveness to monoclonal antibody therapy directed against IL-4, IL-13 and IgE [9–11]. We also estimated the time-related variation in serum periostin levels in adults without asthma, and compared the time-related variations in serum periostin with those of other markers of type-2 dominant asthma, FeNO and blood eosinophils.

## Methods

In this non-experimental cohort observational study we recruited adults aged 18–75 years of age, comprising 16 participants with asthma who were prescribed maintenance ICS and LABA therapy, (asthma group), and 16 participants who neither had a doctor diagnosis of asthma, symptoms of wheeze, or inhaler use in the past 12 months (non-asthma group). Exclusion criteria for both the asthma and non-asthma groups included: chronic bronchitis or COPD, known pregnancy, active (current, or within the three weeks prior to the visit) upper or lower respiratory tract infection, any of the following within the last 3 months; hospital admission, major surgery requiring general anesthetic, dental extractions or root canal procedures and bone fracture and any significant comorbidities or any safety concerns at the investigator’s discretion.

### Study procedures

Participants attended the clinical trials unit (CTU) at Capital and Coast District Health Board (CCDHB Wellington Hospital) for an initial visit (Visit 1) where the following procedures were completed: written informed consent, completion of a general health questionnaire [22], measurement of body mass index (BMI) and training in spirometry technique. Participants then attended the CTU on a second occasion (Visit 2) where blood samples were drawn every 2 h for serum periostin levels and peripheral blood eosinophil counts. Following each blood sample measures of FeNO, forced expiratory volume in 1 s (FEV_1_) and forced vital capacity (FVC) were performed in accordance with ATS criteria [23, 24]. Six measurements were taken over the 10-h period. Participants with asthma also completed the asthma control questionnaire-5 (ACQ-5) and the asthma quality of life questionnaire with standardized activities (AQLQ(S)) [25, 26].

#### Medication use

Participants with asthma were advised to take their regular ICS and LABA treatment in the morning prior to attending the clinic on Visit 2, and not to take any medication during the 10-h study period.

### Study power

The clinically important difference in serum periostin is unknown. We chose a sample size of 16, with 80% power, alpha 5%, based on a paired *t* test, to detect a paired difference of 0.75 standard deviations, for continuous variables, which constitutes in general terms a ‘large’ difference. This same sample size also has good precision for estimation of variance.

### Statistical methods

Simple data descriptions are shown for the variables by asthma status. Serum periostin and FeNO were analyzed on the natural logarithm transformed scale. The exponent of a difference in logarithms can be interpreted as a ratio of geometric means. No transformations were done for other variables.

Mixed linear models were used to compare time zero with subsequent measurement time by asthma groups. The correlation structure for the repeated measures allowed for different correlations between the different measurements on the same participants, the ‘unstructured’ option in the statistical software. Simple unpaired t tests were used to compare the baseline values for asthma versus non-asthma.

A post hoc analysis was undertaken to determine the proportion of adults with asthma that would change classification from ‘high periostin’ to ‘low periostin’, or from ‘low periostin’ to ‘high periostin’ based on the 0800 and 1800 h periostin levels, utilizing the proposed periostin cut point of 50 ng/mL, used to determine responsiveness to monoclonal antibody therapies [9, 10].

SAS version 9.3 was used.

## Results

### Participant characteristics

**Fig. 1:**
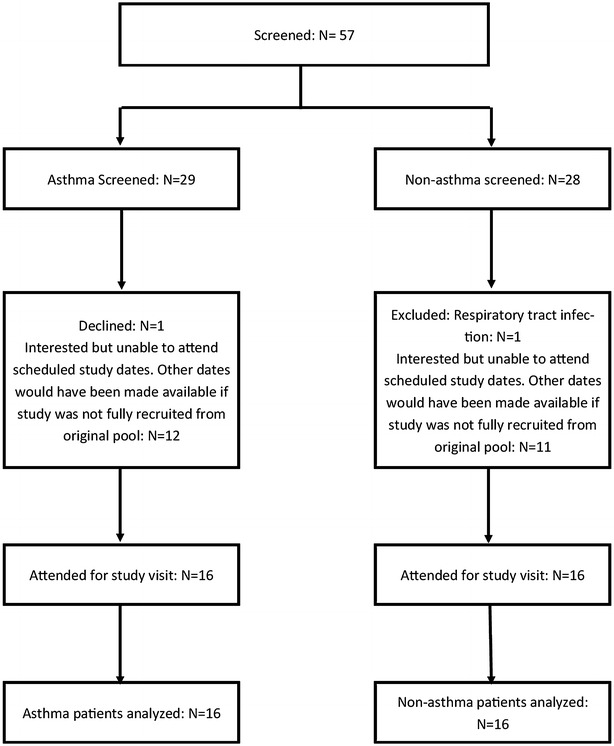
Flow of participants through study

The flow of participants in the study is shown in Fig. 1. Baseline characteristics for asthma and non-asthma participants are shown in Table 1. All asthma participants were prescribed maintenance ICS/LABA inhaler therapy with a mean daily dose of 489 µg/day of fluticasone propionate (FP) or equivalent as the ICS component. The non-asthma participants were younger and had a lower BMI than the asthma participants. There were two current smokers, both of whom had asthma.

**Table 1:** Baseline characteristics of participants

Variable N = 16	Mean (SD)	Median (IQR)	Min to max
*Asthma*			
Age (years)	45.4 (19.7)	47 (25 to 63.5)	19–73
BMI (kg/m^2^)	32.2 (5.3)	33.4 (28.8–35.5)	20.9–42.2
FEV_1_ % predicted^a^	90.5 (18.1)	96.6 (81.9–102.9)	50.6–111.6
FEV1/FVC ratio^b^	0.82 (0.04)	0.81 (0.79–0.85)	0.77–0.89
Mean ICS dose^c^	489 (105.3)	500 (500–500)	200–500
Eosinophil count (×10^9^/L)^d^	0.31 (0.20)	0.25 (0.20–0.35)	0–0.7
FeNO (ppb)	26.1 (13.3)	23.5 (16–36)	7–55
Periostin (ng/mL)	53.5 (13.6)	51.7 (41.5–63.7)	33.9–76.2
ACQ-5^e^	1.3 (1.2)	1.2 (0.4–1.4)	0–4.0
AQLQ(S)	5.71 (1.22)	6.05 (5.5–6.35)	2.8–7.0

Categorical variables	Number (%)		
ACQ ≤ 0.75	5 (31)		
ACQ ≥ 1.50	3 (19)		
ACQ > 0.75 to <1.50	8 (50)		
Atopy^f^ (yes)	13 (81)		
Female	12 (75)		
Caucasian	12 (75)		
Asian	1 (6)		
Other	3 (19)		

Variable N = 16	Mean (SD)	Median (IQR)	Min to max
*Non*-*asthma*			
Age (years)	28.1 (12.7)	23 (21–30.5)	20–68
BMI (kg/m^2^)	23.8 (2.8)	22.8 (22.1–27.0)	19.7–28.3
FEV_1_ % predicted^a^	97.9 (10.5)	96.3 (90.8–105.7)	80.5–120.9
FEV_1_/FVC ratio^b^	0.85 (0.03)	0.86 (0.85–0.88)	0.77–0.88
Eosinophil count (×10^9^/L)	0.19 (0.11)	0.20 (0.10–0.25)	0.10–0.50
FeNO (ppm)	22.3 (14.1)	19.5 (15.5–24.5)	6.0–68.0
Periostin (ng/mL)	50.5 (13.0)	49.4 (42.5–62.7)	28.6–70.5

Categorical variables	Number (%)		
Atopy (yes)	5 (31)		
Female	11 (69)		
Caucasian	13 (81)		
Other	3 (19)		

*SD* standard deviation, *IQR* interquartile ratio, *BMI* body mass index, *ICS* inhaled corticosteroid, *FeNO* fraction of exhaled nitric oxide

^a^FEV_1_ post-bronchodilator, expressed as % of normal predicted values

^b^FEV_1_/FVC ratio, pre-bronchodilator

^c^ICS daily dose expressed as fluticasone propionate equivalent (microg/day)

^d^Eosinophil laboratory reference range: 0.0–0.5 × 10^9^/L, (measured in increments of 0.1)

^e^ACQ-5: asthma control questionnaire-5, a score >1.5 indicates uncontrolled asthma [26]

^f^Atopy: a history of eczema and/or seasonal rhinitis

There was no statistically significant difference in periostin levels between the asthma and non-asthma groups at baseline (0800); difference (95% CI) in logarithm periostin 0.058 (−0.13 to 0.25), P = 0.54. This is equivalent to a mean ratio of serum periostin levels in the asthma versus non-asthma group of 1.06 (0.88–1.28). There was no statistically significant difference in FeNO levels between the asthma and non-asthma groups at baseline (0800); difference (95% CI) in logarithm FeNO 0.16 (−0.25 to 0.56), P = 0.43. This is equivalent to a mean ratio of FeNO levels in the asthma versus non-asthma group of 1.17 (0.78–1.76). There was weak evidence of a higher mean blood eosinophil count, 0.31 × 10^9^/L in the asthma group, compared to 0.19 × 10^9^/L in non-asthma, difference (95% CI), 0.11 (−0.005 to 0.23), P = 0.06.

### Day-time changes in asthma

**Fig. 2:**
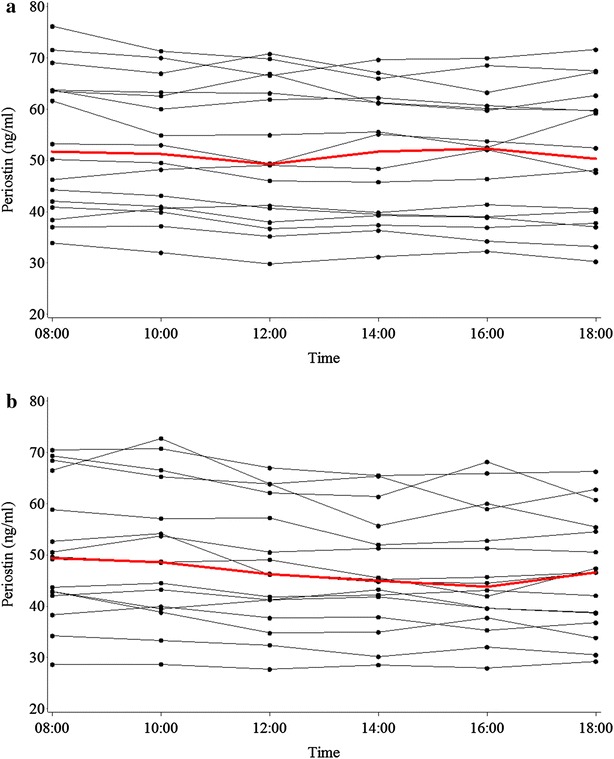
Individual joined line plots for Periostin. *Panel* **a** shows the asthma group, *Panel* **b** shows the non-asthma group. The *bold red line* indicates the median periostin value

The serum periostin level decreased from a mean of 53.5 ng/mL, at 0800 h, to 50.9 ng/mL at 1800 h. There was strong evidence, overall P < 0.001, that the means by time were different, (Table 2; Fig. 2a; Additional file 1: Table S1, Figure S1). Compared with baseline, the log periostin was significantly lower from the 4-h (1200 h) to the 10-h (1800 h) time points, and the size of the difference remained constant from the 4-h time point. The ratio of geometric mean serum periostin compared to baseline was between 0.95 and 0.98.

**Table 2:** Serum periostin (ng/mL) levels at time points during study

Time points	Periostin					
	Mean (SD)	Median (IQR)	Min to max	Difference from baseline mean (SD)	Difference (95% CI) from baseline log periostin^a^	Overall P value <0.001
*Asthma*						
0 (0800)	53.5 (13.6)	51.7 (41.5–63.7)	33.9–76.2			
2 (1000)	52.1 (12.5)	51.3 (40.8–63.0)	32–71.4	−1.4 (2.2)	−0.02 (−0.05 to 0.003)	0.08
4 (1200)	51.3 (13.7)	49.3 (39.3–64.8)	29.9–70.8	−2.2 (3.3)	−0.05 (−0.07 to −0.02)	<0.001
6 (1400)	51.0 (12.7)	51.7 (39.4–61.7)	31.2–69.6	−2.5 (2.5)	−0.05 (−0.07 to −0.02)	<0.001
8 (1600)	50.6 (12.4)	52.3 (38.9–60.4)	32.3–70.0	−2.9 (3.7)	−0.05 (−0.08 to −0.03)	<0.001
10 (1800)	50.9 (13.4)	50.3 (38.9–61.2)	30.3–71.6	−2.6 (2.6)	−0.05 (−0.08 to −0.03)	<0.001
*Non*-*asthma*						
0 (0800)	50.5 (13.0)	49.4 (42.5–62.7)	28.6–70.5			
2 (1000)	50.3 (13.4)	48.6 (39.7–61.2)	28.7–72.7	−0.2 (2.7)	−0.006 (−0.04 to 0.02)	0.71
4 (1200)	47.7 (12.2)	46.3 (39.5–59.7)	27.7–67.0	−2.8 (2.5)	−0.06 (−0.09 to −0.03)	<0.001
6 (1400)	46.6 (11.3)	45.0 (39.8–53.8)	28.6–65.5	−3.9 (3.6)	−0.08 (−0.11 to −0.05)	<0.001
8 (1600)	46.5 (11.9)	43.8 (38.6–55.9)	27.9–68.2	−4.0 (2.9)	−0.08 (−0.11 to −0.05)	<0.001
10 (1800)	46.2 (11.5)	46.6 (37.7–55.0)	29.2–66.3	−4.2 (3.2)	−0.08 (−0.11 to −0.06)	<0.001

*SD* standard deviation, *IQR* interquartile range, *CI* confidence interval

^a^Difference from baseline is estimated from the mixed-effects linear model

In a post hoc analysis of the asthma group, there was a change in classification from ‘high periostin’ to ‘low periostin’, based on the 0800 and 1800 h periostin levels, utilizing the proposed cut point of 50 ng/mL in 1/16 participants (50.3 and 48.2 ng/mL at 0800 and 1800 h, respectively) (Additional file 1: Table S1). There was no change in classification from ‘low periostin’ to ‘high periostin’, using the same criteria.

**Fig. 3:**
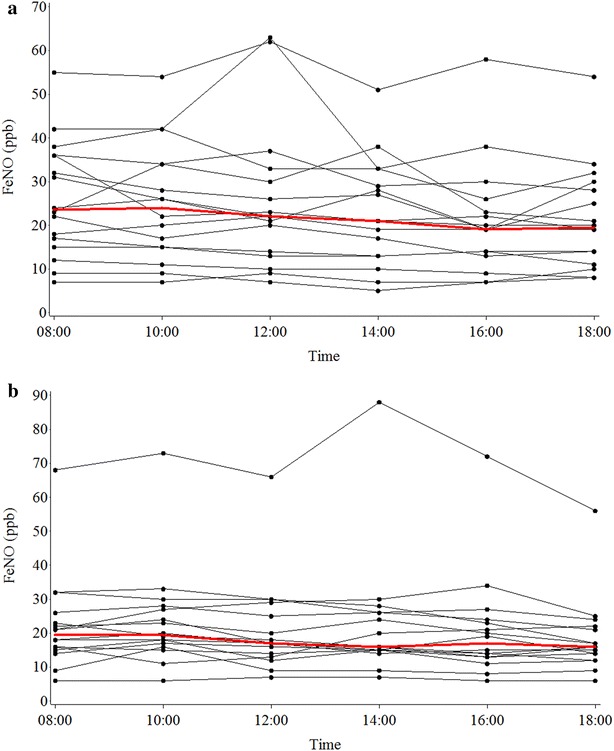
Individual joined line plots for the fraction of exhaled nitric oxide (FeNO). *Panel* **a** shows the asthma group and *Panel* **b** shows the non-asthma group. The *bold red line* indicates the median FeNO value

The FeNO decreased during the day from a peak mean of 26.1 ppb at 0800 h to lowest level of 21.7 ppb at 1800 h. There was strong evidence, overall P < 0.001, that the means by time were different, (Table 3; Fig. 3a, Additional file 1: Table S2, Figure S2). Compared with baseline, the log FeNO was significantly lower from the 6-h (1400 h) to the 10-h (1800 h) time points. The size of the difference from the 6-h time point ranged from an estimate difference (95% CI) of −0.16 (−0.26 to −0.05) to −0.22 (−0.33 to −0.12). The ratio of geometric mean FeNO compared to baseline was between 0.80 and 0.96.

**Table 3:** FeNO (ppb) levels at time points during study

Time points	FeNO					
	Mean (SD)	Median (IQR)	Min to max	Difference from baseline mean (SD)	Difference (95% CI) from baseline log FeNO^a^	Overall P value <0.001
*Asthma*						
0 (0800)	26.1 (13.3)	23.5 (16–36)	7.0–55.0		–	–
2 (1000)	25.1 (13.3)	24.0 (15–34)	7.0–54.0	−1.0 (5.2)	−0.04 (−0.15 to 0.07)	0.44
4 (1200)	25.8 (16.7)	22.0 (13.5–31.5)	7.0–63.0	−0.3 (8.8)	−0.05 (−0.16 to 0.05)	0.32
6 (1400)	22.8 (12.4)	21.0 (13–31)	5.0–51.0	−3.3 (6.1)	−0.16 (−0.26 to −0.05)	0.005
8 (1600)	21.3 (12.9)	19.0 (13.5–24.5)	7.0–58.0	−4.9 (5.7)	−0.22 (−0.33 to −0.12)	<0.001
10 (1800)	21.7 (12.1)	19.5 (12.5–29)	8.0–54.0	−4.4 (4.5)	−0.18 (−0.29 to −0.07)	0.001
*Non*-*asthma*						
0 (0800)	22.3 (14.1)	19.5 (15.5–24.5)	6.0–68.0		–	–
2 (1000)	23.6 (14.9)	19.5 (16.5–27.5)	6.0–73.0	1.4 (2.2)	0.06 (−0.05 to 0.18)	0.30
4 (1200)	21.3 (13.9)	17.0 (13.5–27.0)	7.0–66.0	−1.1 (3.0)	−0.05 (−0.17 to 0.06)	0.38
6 (1400)	22.8 (18.6)	16.0 (15.0–26.0)	7.0–88.0	0.4 (6.6)	−0.02 (−0.14 to 0.09)	0.72
8 (1600)	20.9 (15.4)	17.0 (13.0–23.5)	6.0–72.0	−1.4 (5.9)	−0.10 (−0.22 to 0.01)	0.09
10 (1800)	18.5 (11.2)	16.0 (13.0–21.5)	6.0–56.0	−3.8 (5.5)	−0.17 (−0.28 to −0.05)	0.005

*SD* standard deviation, *IQR* interquartile range, *CI* confidence interval, *FeNO* fraction of exhaled nitric oxide

^a^Difference from baseline is estimated from the mixed-effects linear model

There was some evidence, P = 0.02, that the mean blood eosinophil count differed by time during the 10-h period of the study (Table 4) but none of the individual comparisons with baseline were statistically significant.

**Table 4:** Blood eosinophil (×10^9^/L) levels at time points during study

Time points	Blood eosinophil				
	Mean (SD)	Median (IQR)	Min to max	Difference (95% CI) from baseline^a^	Overall P value = 0.015
*Asthma*					
0 (0800)	0.31 (0.20)	0.25 (0.20–0.35)	0–0.7	–	–
2 (1000)	0.31 (0.20)	0.20 (0.20–0.40)	0.10–0.7	0.006 (−0.03 to 0.04)	0.71
4 (1200)	0.29 (0.20)	0.20 (0.15–0.40)	0.10–0.7	−0.02 (−0.05 to 0.01)	0.26
6 (1400)	0.29 (0.21)	0.2 (0.1–0.4)	0.1–0.8	−0.02 (−0.05 to 0.01)	0.26
8 (1600)	0.31 (0.19)	0.25 (0.20–0.40)	0.1–0.7	0 (−0.03 to 0.03)	0.99
10 (1800) N = 15	0.34 (0.21)	0.30 (0.20–0.40)	0.1–0.8	0.02 (−0.01 to 0.06)	0.20
*Non*-*asthma*					
0 (0800)	0.19 (0.11)	0.20 (0.10–0.25)	0.10–0.50	–	–
2 (1000)	0.16 (0.10)	0.10 (0.10–0.20)	0.20–0.50	−0.04 (−0.07 to −0.01)	0.023
4 (1200) N = 15	0.13 (0.10)	0.10 (0.10–0.20)	0–0.4	−0.06 (−0.09 to −0.02)	0.001
6 (1400)	0.14 (0.10)	0.10 (0.10–0.20)	0.0–0.3	−0.06 (−0.09 to −0.02)	<0.001
8 (1600)	0.15 (0.08)	0.10 (0.10–0.20)	0.10–0.30	−0.04 (−0.08 to −0.01)	0.008
10 (1800) N = 15	0.17 (0.09)	0.10 (0.10–0.20)	0.10–0.40	−0.03 (−0.06 to 0.01)	0.13

*SD* standard deviation, *IQR* interquartile range, *CI* confidence interval

^a^Difference from baseline is estimated from the mixed-effects linear model

FEV_1_ % predicted progressively decreased during the day from 90.5% at 0800 h to 85.8% at 1800 h (Additional file 1: Table S3). No participant received SABA for symptom relief during the 10-h period of the study.

### Day-time changes in non-asthma

Serum periostin level progressively decreased during the day from a mean of 50.5 ng/mL at 0800 h to 46.2 ng/mL at 1800 h. There was strong evidence, P < 0.001, that the means by time were different, (Table 2; Fig. 2b; Additional file 1: Table S4, Figure S3). The magnitude of the difference compared with baseline was, as for the asthma group, stable from the 4-h (1200 h) to the 10-h (1800 h) time points. The ratio of geometric mean serum periostin compared to baseline was between 0.92 and 1.00.

FeNO progressively decreased during the day from 22.3 ppb at 0800 h to 18.5 ppb at 1800 h. There was strong evidence, (P = 0.004), that the means by time were different, (Table 3; Fig. 3b; Additional file 1: Table S5, Figure S4). Compared with baseline, the log FeNO was significantly lower from the 10-h (1800 h) time point with an estimated difference (95% CI) of 0.17 (−0.28 to −0.05; P = 0.005). The ratio of geometric mean FeNO compared to baseline was between 0.84 and 0.98.

Blood eosinophil count progressively decreased during the day from a mean of 0.19 × 10^9^/L at 0800 h to a nadir of 0.13 × 10^9^/L. There was strong evidence that the means were different by time, (P < 0.001) (Table 4). Compared with baseline, the blood eosinophil count was significantly lower at the 2-, 4-, 6- and 8-h time points with estimated differences (95% CI) ranging from −0.06 (−0.09 to −0.02) to −0.04 (−0.08 to −0.01).

## Discussion

This study shows that serum periostin levels vary throughout the day in adults with and without asthma, with higher levels in the morning in both groups. The magnitude of the variation was small, suggesting that the time that periostin levels are measured is unlikely to influence treatment decisions if predicting responsiveness to, or eligibility for monoclonal antibody therapy directed against IL-4Ra, IL-13, IgE or other components of type-2 inflammation in asthma.

The median baseline values of 51.7 and 49.4 ng/mL in the asthma and non-asthma groups respectively are similar to the median levels of 50.1 ng/mL in a population without asthma or COPD [12], 53.7 ng/mL in a random adult population with a diagnosis of asthma [13], and 50.2 ng/mL in adults with moderate to severe asthma inadequately controlled despite ICS therapy [9]. Interpretation of periostin levels might be confounded by the observation that initiation of ICS therapy may result in a modest reduction in serum periostin by a mean of 5.3 ng/mL [13]. Together, these findings suggest that serum periostin is not a measure which can differentiate patients with asthma across a range of severity from a population without asthma.

The daytime variation we have found with serum periostin levels is similar to that reported previously for the type 2-related biomarkers of sputum and blood eosinophils [14–18] and FeNO [19–21]. In allergic subjects with mild asthma, sputum eosinophils are about two-fold higher at 0700 h than at 1600 h [14]. Additionally, the early morning increase in sputum eosinophils correlated with enhanced airway obstruction and reversibility, suggesting that airway recruitment of eosinophils might contribute to circadian variations in lung function in patients with asthma [14].

In adults with mild asthma, blood eosinophil counts are about 25% higher at 0400 h than at 1600 h [15]. It has been observed that the circadian change in blood eosinophils and lung function appear to fall into a continuous range, suggesting that day/night variations in airways inflammation and lung function occur as a continuum, rather than as an all or nothing phenomenon [15]. In allergic subjects with moderately severe asthma, a circadian variation in blood eosinophil counts was also observed with peak values overnight [16]. In healthy subjects, blood eosinophil counts may also vary diurnally, being lowest in the morning and highest at night, correlating inversely to blood cortisol levels [17]. The demonstration of daytime variation in the non-asthma, but not the asthma group in our study is likely to reflect the lack of power, low sensitivity of the automated measurement of blood eosinophil levels to increments of 0.1 per 10^9^/L, and possibly the effect of the maintenance treatment with ICS in all asthma participants.

In asthma, there is a variable degree of diurnal variation in FeNO, which is greatest in uncontrolled disease and serves as a predictor of risk of future exacerbations [19–21]. The mean diurnal FeNO variation, measured as the difference in morning (0700–1000 h) from evening (1800–2100 h) levels over a two-week period, was 15.6 ppb in uncontrolled asthma subjects compared with 8.2 ppb in stable controlled asthma, and 6.1 ppb in healthy subjects [21]. In another study of adults with asthma, morning FeNO levels were reported to be 14% higher than evening levels [19]. In our study we observed a mean difference in FeNO between 0800 and 1800 h on a single day of 4.4 ppb in controlled asthma and 3.8 ppb in non-asthma. Finally, the lesser variability, based on the ratio of geometric mean values compared to baseline, of serum periostin levels compared with FeNO levels in our study is consistent with previous observations that there is a lesser intra-patient variability in periostin levels compared with FeNO [9].

The clinical relevance of our findings is illustrated by the post hoc analysis in which we determined that only one in 16 asthma participants changed their periostin classification between ‘high periostin’ and ‘low periostin’, based on the 0800 and 1800 h levels, utilizing the proposed periostin cut point of 50 ng/mL, used to determine responsiveness to monoclonal antibody therapy directed against IL-13 [9], and IgE [10]. Thus daytime variation of periostin is unlikely to be an important consideration using the 50 ng/mL cut point. However, the validity of the cut-point as a predictor of responsiveness will need to be confirmed in future studies utilizing the same assay and a cut-off needs to be established if other assays are used. Although the used assay has a very good precision around the cut-point of 50 ng/mL (repeatability CV 0.9–1.5%, intermediate precision CV 1.2–1.7% and reproducibility CV 1.7–3.1%), a degree of error of misclassification cannot be excluded It should also be noted that the assay used has a reproducibility CV of 1.7–3.1% around the 50 ng/mL cut point of 1.7–3.1%, introducing a degree of reproducibility error when taking repeat samples [27].

There are a number of methodological limitations relevant to the interpretation of the study findings. No adjustment has been made for multiple statistical testing, thus the findings should be considered illustrative. The findings are generalizable to Caucasian adults with asthma on regular ICS and LABA treatment, representing GINA Step 3 and 4 therapy [28], but generalizability to other ethnicities is less certain. ICS reduces serum periostin levels [13], FeNO [29–31], and blood eosinophil counts [16], however the effect of ICS on the circadian rhythm of periostin is not known. Periostin levels are unlikely to have been influenced by the presence or absence of atopy in either the asthma or non-asthma group [12, 13], but It is possible that serum periostin levels may show a greater magnitude of circadian variability in a population in which there is a greater proportion with uncontrolled disease, similar to an effect shown with FeNO [21]. In this sample there were no participants on chronic oral corticosteroids however this is a group that might be more likely to be considered for monoclonal antibody therapy regardless of serum periostin levels. The use of LABA therapy prior to the baseline morning measurements and no subsequent medication use during the period of the study, is unlikely to have influenced the periostin or FeNO levels, but may have resulted in the gradual reduction in FEV_1_ during the study period as the bronchodilator effect of the LABA wore off.

## Conclusions

We conclude that there is day-time variation of serum periostin in adults with asthma receiving maintenance ICS and LABA therapy, with higher levels in the morning. The magnitude of the variation in serum periostin levels is of uncertain clinical significance, however, the time of day at which the blood sample is drawn was unlikely to influence treatment decisions if a specific serum periostin level was used to predict treatment responsiveness in the asthma group.

## Additional file

**Additional file 1.** Additional figures and tables.

## Abbreviations

COPD: chronic obstructive pulmonary disease
FEV_1_: forced expiratory volume in 1 s
FeNO: fraction of exhaled nitric oxide
GERD: gastro-esophageal reflux disease
ICS: inhaled corticosteroids
IQR: interquartile range
IL: interleukin
IgE: immunoglobulin E
LABA: long-acting beta-agonist
SABA: short-acting beta-agonist
YKL-40: human cartilage glycoprotein -39
