# Supplementary material for: Day-time variation of serum periostin in asthmatic adults treated with ICS/LABA and adults without asthma
Source: Allergy Asthma Clin Immunol. 2017 Feb 8;13:8. doi: 10.1186/s13223-017-0182-0 (PMC5299725; doi:10.1186/s13223-017-0182-0)

**DAY-Time VARIATION OF SERUM Periostin IN asthmatic ADULTS treated with ICS/LABA and adults without asthma**

**ONLINE SUPPLEMENT**

^1,2^Rachel Caswell-Smith, ^1^Terrianne Cripps, ^1^Thom Charles ^1,3^Alexander Hosking, ^4^Meghana Handigol, ^4^Cecile Holweg, ^4^John Matthews, ^1^Mark Holliday, ^1^Corentin Maillot, ^1^James Fingleton ^1,5^Mark Weatherall, ^1,2,6^Irene Braithwaite, ^1,2,6^Richard Beasley

On behalf of the Periostin Study Team

^1^Medical Research Institute of New Zealand, Wellington, New Zealand

^2^Victoria University of Wellington, Wellington, New Zealand

^3^University of Auckland, Auckland, New Zealand

^4^Genentech Inc, San Francisco, California, USA

^5^University of Otago, Wellington, New Zealand

^6^Capital & Coast District Health Board, Wellington, New Zealand

**EXCLUSION CRITERIA**

Exclusion criteria for both the asthma and non-asthma groups included:

- chronic bronchitis or COPD,
- known pregnancy,
- active (current, or within the three weeks prior to the visit) upper or lower respiratory tract infection,
- any of the following within the last 3 months; hospital admission, major surgery requiring general anesthetic, dental extractions or root canal procedures and bone fracture
- any significant comorbidities or any safety concerns at the investigator’s discretion.

**DESCRIPTION OF PROCEDURES**

***General Health Questionnaire:***

The general health questionnaire was used to obtain the current health status of participants, including a history of cancer, cardiovascular disease, gastro-esophageal reflux (GERD), rhinitis, sinusitis, and eczema (Appendix 1). The medical history was obtained using questions drawn from a series of validated questionnaires, with co-morbidities recorded using questions from the American Thoracic Society (ATS) Division of Lung Diseases-78 (DLD-78) questionnaire [21].

***Respiratory Health Questionnaires:***

The AQLQ(S) is a validated 32 question self-administered questionnaire which was used to obtain an assessment of the impact of asthma in the two weeks prior to Visit 2 [22].

The ACQ-5 is a validated five question self-administered questionnaire that was used to obtain information about control of asthma in the one week prior to Visit 2 [23]. These questionnaires were administered to the asthma group only.

***Lung function and FeNO:***

Spirometry was performed with measurement of FEV_1_ and FVC using a Masterscreen Pneumo (Masterscreen Version 2.0, Carefusion, Leibnizstrasse Hoechberg, Germany) in accordance with ATS guidelines [24]. FeNO was also determined according to ATS guidelines [25] with an online nitric oxide monitor (NiOX, Aerocrine AB, Solna, Sweden).

***Bloods:***

Full blood count and differential (Sysmex platform, Mundelein, USA) were performed immediately. Blood eosinophil counts were measured to one decimal place, expressed as 10^9^/L. A further blood sample was centrifuged and serum aliquots stored at -80°C, prior to analysis of serum periostin. Serum periostin levels were determined using the clinical trial version of the Elecsys® Periostin Immunoassay (Roche Diagnostics, Penzberg, Germany). The assay is an automated electrochemiluminescence immunoassay, based on the sandwich principle utilizing the same antibodies reported by Jia et al [6].

**Table S1. Periostin Data in Individual Participants with Asthma**

| **Periostin**  **Baseline**  **(ng/ml)** | **Periostin**  **2 hours**  **(ng/ml)** | **Periostin**  **4 hours**  **(ng/ml)** | **Periostin**  **6 hours**  **(ng/ml)** | **Periostin**  **8 hours**  **(ng/ml)** | **Periostin**  **10 hours**  **(ng/ml)** |
| --- | --- | --- | --- | --- | --- |
| 76.16 | 71.36 | 69.83 | 65.99 | 68.55 | 67.43 |
| 63.7 | 60.03 | 61.92 | 62.2 | 60.66 | 59.61 |
| 46.28 | 48.2 | 49.09 | 48.39 | 52.07 | 47.64 |
| 33.89 | 32 | 29.85 | 31.16 | 32.25 | 30.25 |
| 42.03 | 40.95 | 37.92 | 39.3 | 38.85 | 36.99 |
| 69.05 | 66.95 | 70.79 | 67.11 | 63.24 | 67.24 |
| 40.87 | 39.9 | 36.68 | 37.38 | 36.91 | 37.79 |
| 44.31 | 43.12 | 40.7 | 39.48 | 38.99 | 40.02 |
| 37.02 | 37.16 | 35.23 | 36.33 | 34.22 | 33.16 |
| 53.22 | 52.99 | 49.42 | 55.1 | 53.75 | 52.4 |
| 71.6 | 70 | 66.59 | 69.64 | 69.97 | 71.61 |
| 63.68 | 63.29 | 63.1 | 61.28 | 60.11 | 59.79 |
| 61.63 | 54.9 | 54.94 | 55.54 | 52.54 | 59.2 |
| 50.26 | 49.53 | 46.06 | 45.8 | 46.39 | 48.15 |
| 38.39 | 40.63 | 41.18 | 39.81 | 41.39 | 40.51 |
| 63.52 | 62.61 | 66.93 | 61.16 | 59.75 | 62.65 |

Participant highlighted would change classification from ‘high Periostin’ to ‘low Periostin’ based on the baseline and 10 hour periostin levels, utilizing the proposed periostin cut point of 50 ng/ml.

**Table S2. FeNO Data in Individual Participants with Asthma**

| **FeNO**  **Baseline**  **(ppb)** | **FeNO**  **2 hours**  **(ppb)** | **FeNO**  **4 hours**  **(ppb)** | **FeNO**  **6 hours**  **(ppb)** | **FeNO**  **8 hours**  **(ppb)** | **FeNO**  **10 hours**  **(ppb)** |
| --- | --- | --- | --- | --- | --- |
| 55 | 54 | 62 | 51 | 58 | 54 |
| 7 | 7 | 9 | 7 | 7 | 8 |
| 36 | 22 | 23 | 21 | 20 | 20 |
| 31 | 26 | 21 | 28 | 19 | 30 |
| 18 | 20 | 22 | 19 | 19 | 19 |
| 15 | 15 | 14 | 13 | 14 | 11 |
| 36 | 34 | 37 | 29 | 30 | 28 |
| 32 | 28 | 26 | 27 | 19 | 25 |
| 42 | 42 | 33 | 33 | 38 | 34 |
| 9 | 9 | 7 | 5 | 7 | 10 |
| 38 | 42 | 63 | 33 | 26 | 32 |
| 22 | 17 | 20 | 17 | 13 | 14 |
| 17 | 15 | 13 | 13 | 14 | 14 |
| 12 | 11 | 10 | 10 | 9 | 8 |
| 23 | 34 | 30 | 38 | 23 | 21 |
| 24 | 26 | 22 | 21 | 22 | 19 |

**Table S3. Respiratory data in Participants with asthma**

| **N=16 unless stated** | **Mean (SD)** | **Median (IQR)** | **Min to Max** |
| --- | --- | --- | --- |
| FEV1 |  |  |  |
| 0 | 2.75 (1.0) | 2.51 (2.01 to 3.44) | 1.28 to 4.98 |
| 2 | 2.72 (1.1) | 2.55 (1.88 to 3.40) | 1.14 to 5.04 |
| 4 | 2.71 (1.08) | 2.51 (1.87 to 3.42) | 1.14 to 5.05 |
| 6 | 2.69 (1.05) | 2.56 (1.84 to 3.39) | 1.2 to 5.05 |
| 8 | 2.65 (1.04) | 2.54 (1.82 to 3.30) | 1.25 to 4.98 |
| 10 | 2.65 (1.05) | 2.54 (1.80 to 3.34) | 1.26 to 4.99 |
| FEV1 % |  |  |  |
| 0 | 90.5 (18.1) | 96.6 (81.9 to 102.9) | 50.6 to 111.6 |
| 2 | 89.3 (19.8) | 92.3 (80.7 to 105.1) | 45.1 to 113.4 |
| 4 | 88.8 (20.3) | 91.6 (78.0 to 106.6) | 45.1 to 111.2 |
| 6 | 88.4 (19.5) | 89.6 (80.9 to 104.2) | 47.5 to 112.5 |
| 8 | 86.7 (18.7) | 88.3 (75.7 to 103.2) | 47.7 to 106.8 |
| 10 | 85.8 (19.7) | 87.0 (73.2 to 104.4) | 49.8 to 111.2 |
| FEV1/FVC |  |  |  |
| 0 | 0.73 (0.12) | 0.74 (0.71 to 0.80) | 0.34 to 0.89 |
| 2 | 0.73 (0.12) | 0.74 (0.68 to 0.81) | 0.37 to 0.89 |
| 4 | 0.73 (0.12) | 0.76 (0.67 to 0.80) | 0.37 to 0.90 |
| 6 | 0.74 (0.12) | 0.76 (0.69 to 0.81) | 0.37 to 0.89 |
| 8 | 0.73 (0.12) | 0.75 (0.67 to 0.80) | 0.38 to 0.89 |
| 10 | 0.73 (0.13) | 0.76 (0.65 to 0.81) | 0.38 to 0.88 |
| FVC |  |  |  |
| 0 | 3.75 (1.12) | 3.70 (2.99 to 4.49) | 1.91 to 5.78 |
| 2 | 3.69 (1.14) | 3.67 (2.97 to 4.35) | 1.89 to 5.73 |
| 4 | 3.67 (1.15) | 3.52 (2.88 to 4.35) | 1.91 to 5.91 |
| 6 | 3.62 (1.12) | 3.43 (2.93 to 4.27) | 1.88 to 5.68 |
| 8 | 3.60 (1.13) | 3.47 (2.84 to 4.26) | 1.93 to 5.80 |
| 10 | 3.60 (1.12) | 3.50 (2.75 to 4.27) | 1.91 to 5.64 |

**Table S4. Periostin Data in Individual Participants without Asthma**

| **Periostin**  **Baseline**  **(ng/ml)** | **Periostin**  **2 hours**  **(ng/ml)** | **Periostin**  **4 hours**  **(ng/ml)** | **Periostin**  **6 hours**  **(ng/ml)** | **Periostin**  **8 hours**  **(ng/ml)** | **Periostin**  **10 hours**  **(ng/ml)** |
| --- | --- | --- | --- | --- | --- |
| 58.84 | 57.14 | 57.23 | 51.99 | 52.79 | 54.58 |
| 42.09 | 43.22 | 41.25 | 43.27 | 39.56 | 38.62 |
| 50.55 | 53.67 | 50.51 | 51.3 | 51.31 | 50.57 |
| 49.22 | 48.53 | 49.09 | 45.59 | 41.96 | 47.36 |
| 66.51 | 72.73 | 63.88 | 55.66 | 60.01 | 55.41 |
| 70.46 | 70.76 | 66.98 | 65.54 | 65.93 | 66.25 |
| 49.64 | 48.71 | 46.34 | 45.21 | 45.71 | 46.63 |
| 38.3 | 39.91 | 37.69 | 37.87 | 35.32 | 36.77 |
| 68.46 | 65.27 | 63.84 | 65.35 | 58.96 | 62.75 |
| 43.68 | 44.5 | 41.83 | 42.21 | 43.13 | 42.07 |
| 42.81 | 38.78 | 34.77 | 34.92 | 37.71 | 33.83 |
| 28.61 | 28.67 | 27.68 | 28.55 | 27.89 | 29.22 |
| 69.35 | 66.54 | 62.13 | 61.35 | 68.16 | 60.75 |
| 42.88 | 39.44 | 41.23 | 41.82 | 39.57 | 38.75 |
| 34.21 | 33.36 | 32.39 | 30.14 | 32.02 | 30.48 |
| 52.62 | 54.13 | 46.16 | 44.79 | 44.54 | 46.59 |

Participant highlighted would change classification from ‘high Periostin’ to ‘low Periostin’ based on the baseline and 10 hour periostin levels, utilizing the proposed periostin cut point of 50 ng/ml.

**Table S5. FeNO Data in Individual Participants without Asthma**

| **FeNO**  **Baseline**  **(ppb)** | **FeNO**  **2 hours**  **(ppb)** | **FeNO**  **4 hours**  **(ppb)** | **FeNO**  **6 hours**  **(ppb)** | **FeNO**  **8 hours**  **(ppb)** | **FeNO**  **10 hours**  **(ppb)** |
| --- | --- | --- | --- | --- | --- |
| 68 | 73 | 66 | 88 | 72 | 56 |
| 16 | 11 | 13 | 20 | 21 | 22 |
| 22 | 23 | 20 | 24 | 20 | 17 |
| 16 | 15 | 14 | 15 | 19 | 14 |
| 18 | 18 | 17 | 16 | 14 | 16 |
| 32 | 30 | 30 | 28 | 23 | 17 |
| 21 | 27 | 29 | 30 | 34 | 25 |
| 18 | 20 | 17 | 14 | 15 | 15 |
| 21 | 24 | 17 | 15 | 11 | 12 |
| 26 | 28 | 25 | 26 | 24 | 21 |
| 14 | 17 | 16 | 15 | 13 | 14 |
| 15 | 18 | 12 | 15 | 14 | 12 |
| 23 | 19 | 18 | 16 | 13 | 16 |
| 9 | 16 | 9 | 9 | 8 | 9 |
| 6 | 6 | 7 | 7 | 6 | 6 |
| 32 | 33 | 30 | 26 | 27 | 24 |

**Figure Legends**

**Figure S1. Change in individual periostin measures from baseline (Asthma Group)**

**Figure S2. Change in individual FeNOs from baseline (Asthma Group)**

**Figure S3. Change in individual Periostin measures from baseline (Non-Asthma)**

**Figure S4. Change in individual FeNOs from baseline (Non-Asthma)**


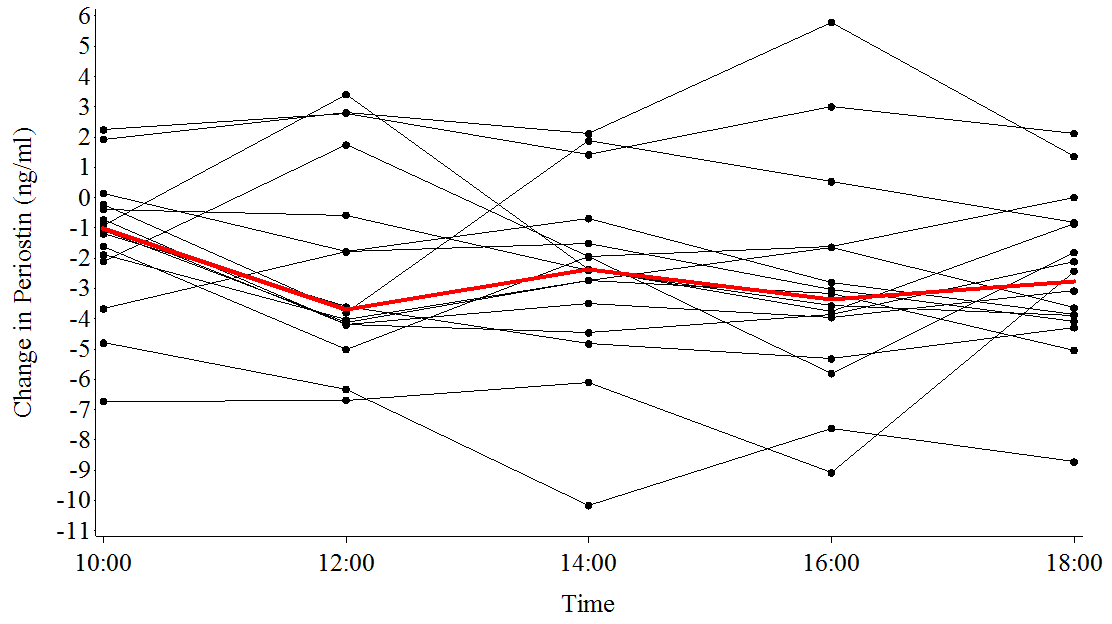


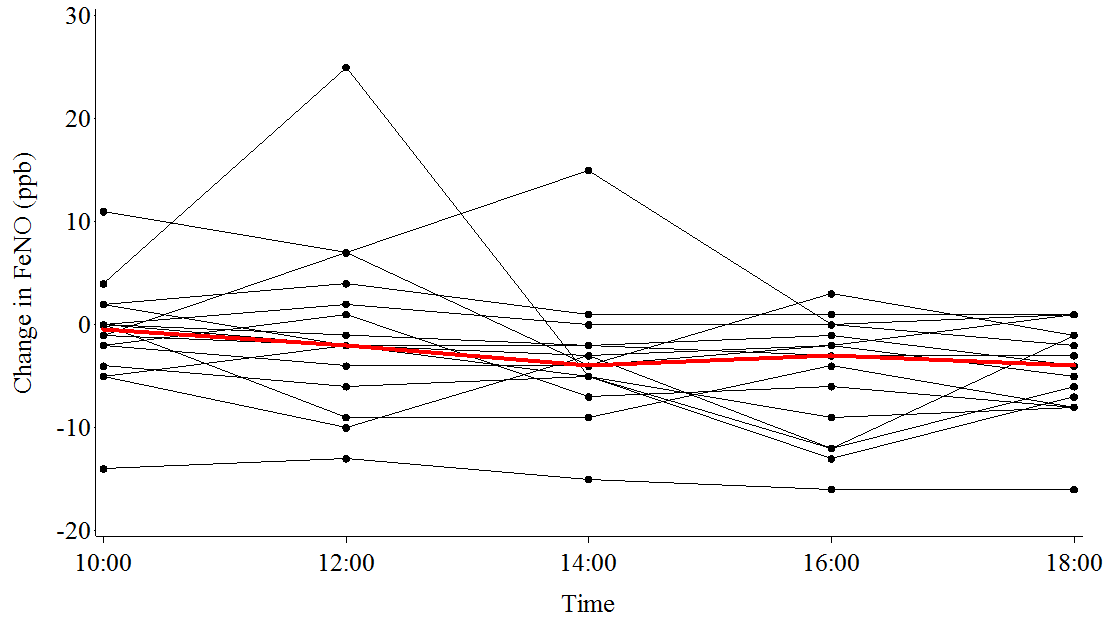


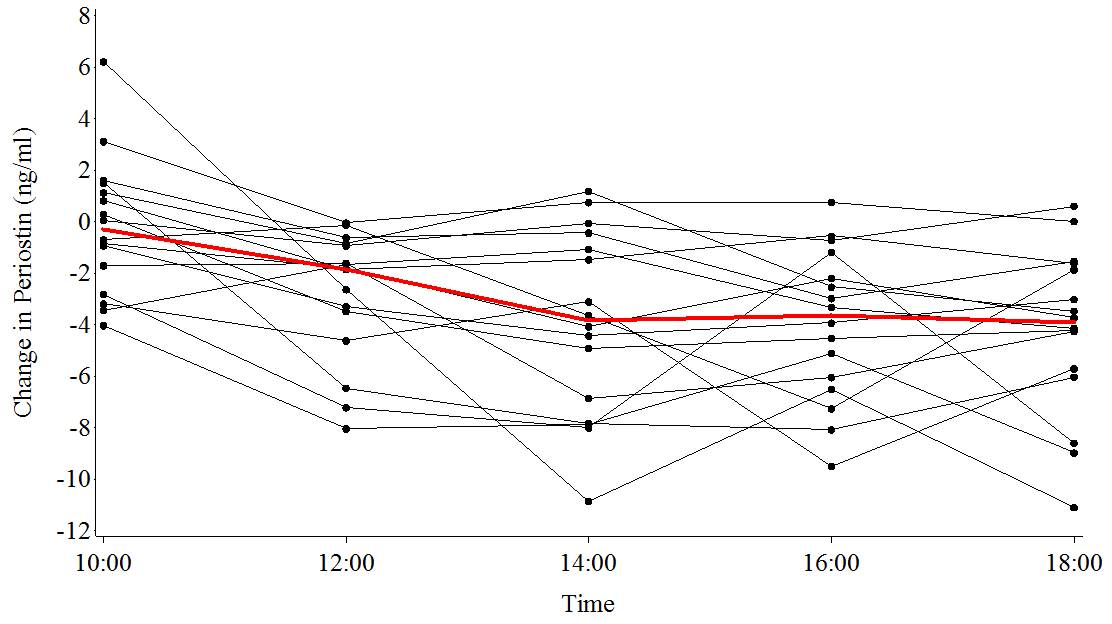


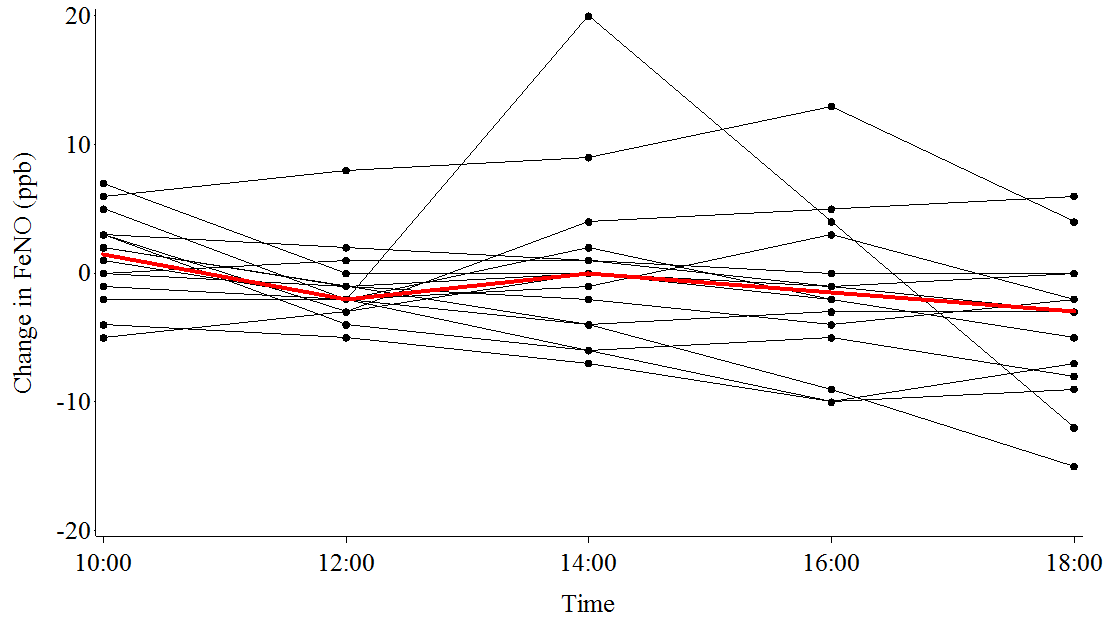

Supplement: Supplementary file 1 — Additional file 1. Additional figures and tables. [file 13223_2017_182_MOESM1_ESM.doc]
